# General-practitioner-centered health care: current results from the implementation of the German model

**DOI:** 10.1038/s41598-026-43163-x

**Published:** 2026-03-09

**Authors:** Ruediger Leutgeb, Gerhard Emmanuel Fuchs, Attila Altiner, Gunter Laux

**Affiliations:** Dept. of General Practice and Health Services Research, Heidelberg University, D-69120 Heidelberg, Germany

**Keywords:** Health care, Medical research

## Abstract

Primary care-centered healthcare models, particularly those led by general practitioners (GPs), are increasingly adopted to address global healthcare challenges including rising costs, fragmented services, and chronic disease burdens. In Germany, the “Hausarztzentrierte Versorgung” (English: General Practitioner-Centered Health Care, GPCHC) program aims to reinforce the role of GPs as care coordinators. Within this study we evaluated data from the implemented German GPCHC model in Baden-Württemberg, a German federal State with about 11 Mio inhabitants and compared outcomes with international benchmarks for strong primary care. The analysis is based on administrative health insurance data of almost two million individuals. We compared patients enrolled in GPCHC with patients receiving regular primary care in 2022 in terms of key indicators of healthcare utilization (GP contacts, uncoordinated consultations with a non-GP-specialist, all-cause hospitalizations, potentially avoidable hospitalizations (PAHs), and prescription of me-too drugs. For patients enrolled in the GPCHC program, consistently favorable outcomes were observed with respect to these key indicators. These findings align with international evidence from strong primary care systems in the Netherlands, the United Kingdom, and Nordic countries. The proposed model presents a scalable framework for strengthening primary care delivery in complex healthcare systems.

## Introduction

General practitioner-centered health care (GPCHC) is increasingly recognized as a foundational approach for addressing the structural challenges of modern health systems. Conceptually grounded in the ten building blocks of high-performing primary care—such as empanelment, team-based care, population health management, and continuity. GPCHC models are designed to enhance care integration, responsiveness, and sustainability^1^.

Over the past 15 years, a growing body of international research has established strong associations between GPCHC models and improved health system performance, particularly in the context of rising costs, care fragmentation, and the global burden of chronic disease^2–4^. The burden of disease refers to the overall impact of health problems on a population or a specific patient, especially how much disability and reduced quality of life it causes. Countries with well-developed GP-centered systems consistently report lower rates of potentially avoidable hospitalizations (PAHs), better chronic disease control, and higher levels of patient satisfaction^5^.

The findings of the QUALICOPC study, which evaluated primary care in thirty-four countries, further underscore these relationships^5^. Systems with institutionalized gatekeeping mechanisms—such as the Netherlands, Denmark, and the United Kingdom—demonstrated high continuity of care, broad scope of services, and superior patient-reported experiences. In the Netherlands, near-universal general practitioner (GP) enrollment and comprehensive care provision were linked to improved coordination and reduced specialist overutilization. Denmark demonstrated high levels of equity and accessibility of after-hours care, while the UK showed solid performance in the areas of patient-centeredness and chronic disease management. These patterns align with international evidence suggesting that robust primary care systems are inversely correlated with health inequities and preventable hospital admissions^6–11^.

In contrast, Germany, where GPCHC was traditionally voluntary and heterogeneous, has seen significant structural development in recent years. The German Association of General Practitioners (“Deutscher Hausärzteverband”) has implemented a nationwide rollout of the GPCHC model, contributing to the gradual resolution of care fragmentation and improving integration across primary care settings^12^.

Recent German cohort studies further support the effectiveness of GPCHC models in improving patient outcomes. An evaluation based on routine data from AOK Baden-Württemberg showed significant reductions in all-cause hospitalizations among participants of structured GPCHC care programs^13^. A large-scale survival analysis revealed lower mortality rates over five years for patients enrolled in GPCHC, particularly among chronically ill and elderly populations^14^. Additionally, it was observed that GPCHC was associated with lower rates of PAHs for nursing home patients^15^.

Longitudinal studies also documented sustained reductions in preventable hospital admissions and improved continuity of care during the COVID-19 pandemic^16^. Moreover, recent pharmacoepidemiologic research linked GP-centered models with a lower prescription prevalence of fall-risk-increasing drugs (FRIDs) in elderly patients, highlighting the system’s role in medication safety^17^.

These findings emphasize the relevance of structured GPCHC for clinical effectiveness, resource efficiency, and patient safety in the German health system.

Building on this evidence base, the present analysis integrates the most recent GPCHC findings with longitudinal data to show robustness and places them within the comparative framework of international high-performing primary care models. We especially focused on GP contacts, uncoordinated consultations with a non-GP-specialist, all-cause hospitalizations, PAHs, and prescription of me-too drugs. In most cases, me-too are “pseudo-innovations” that do not provide additional therapeutic benefit but are more costly than already existing medications. Within the framework of the GPCHC, general practitioners receive targeted training to raise awareness of this issue. As a result, fewer me-too drugs should be prescribed, contributing to more cost-effective pharmacotherapy without compromising therapeutic benefit.

## Methods

### Study design

This observational study utilized routinely collected administrative data from a major statutory health insurer in Germany. The data were originally gathered for the purpose of reimbursing healthcare providers. The insurer, AOK Baden-Wuerttemberg, covers more than 40% of the population in the federal state of Baden-Wuerttemberg (overall about 11 million individuals). The study focused on a one-year observation period spanning from January 1, 2022, to December 31, 2022. The longitudinal analyses focused on a 12-year observation period spanning from January 1 st, 2011, to December 31 st, 2022.

### Study population

Participants were selected according to predefined inclusion criteria:

- Age of 18 years or older.
- Residency in Baden-Wuerttemberg during the study year.
- At least one general practitioner (GP) consultation within the observation period.
- Continuous health insurance coverage with AOK Baden-Wuerttemberg.
- No enrollment in other healthcare agreements (e.g., integrated care contracts).
- No gaps in insurance registration.
- Exclusion criteria comprised death or withdrawal from the GP-centered healthcare (GPCHC) program during the study period. GPCHC participants were directly linked to their designated GP. In contrast, control group patients were retrospectively assigned to the GP whom they consulted in at least 50% of their visits. Control patients for whom no such assignment was possible (approximately 5.9%) were excluded from the analysis. The most frequently consulted physician was considered the patient’s GP. Finally, we excluded patients in regular care with a GP in the CPCHC programme in order to avoid potential spillover effects.

### Programme

The GPCHC programme, established under Paragraph 73b of the German Social Code Book Five (SGB V), is intended to enhance the role of primary care. General practitioners (GPs) participating in the programme receive approximately 40% higher reimbursement per enrolled patient, with no maximum limit. Patients, however, do not receive direct financial incentives for participation.

The programme is characterized by several core elements:

- Timely Access to Services: Participating practices ensure the availability of appropriate infrastructure, regular consultation hours, and modern IT systems, resulting in reduced waiting times and exemption from out-of-pocket costs for medications.
- Holistic Primary Care: GPs are qualified in essential areas of primary care and are required to engage in ongoing professional training.
- Electronic Decision Support Tools: A traffic light–based system supports prescribing practices by promoting cost-effective and evidence-informed medication choices.
- Structured Management of Chronic Diseases: The programme provides organized care pathways for chronic conditions such as diabetes, asthma, chronic obstructive pulmonary disease (COPD), and coronary heart disease.
- GP-Guided Access to Specialist Services: In line with gatekeeping principles, referrals to specialists require GP coordination and oversight.
- Enhanced Care Coordination: Communication and information exchange between providers are improved, particularly in the referral process.
- Quality Improvement Through Data Feedback: Participating GPs engage in quality circles, receiving data-driven feedback on prescribing behavior along with regular updates on evidence-based guidelines.

The programme is voluntary for both, GPs and patients. As a rule, patients do not actively select a primary care physician for enrollment; rather, they are invited by their GP participating in the GPCHC program to also enroll in the GPCHC. As mentioned above, there is a financial incentive for the reimbursement of care provided to GPCHC patients. However, the workload of GPCHC primary care physicians is also higher, as GPCHC patients contact their primary care physician more frequently. In addition, GPCHC physicians are required to offer regular evening consultation hours for their GPCHC patients. Furthermore, in contrast to colleagues who do not participate in the GPCHC program, GPCHC GPs are obligated to take part regularly in additional continuing medical education activities, such as quality circles. GPCHC patients benefit from more effectively coordinated care provided by their primary care physician, who —also through evening consultation hours exclusively available to GPCHC patients— has more time available for them. In addition, copayments are waived for patients for specific medications.

Apart from these differences, patient care is highly similar for both patient groups. Consequently, a considerable number of patients are treated by a CPCHC GP despite not being enrolled in the CPCHC program themselves. However, these patients – as mentioned above – were excluded from our analyses in order to avoid potential spillover effects.

### Key indicators of healthcare utilization

GP contacts, uncoordinated consultations with a non-GP-specialist, and all-cause hospitalizations were directly available within the routine data set. PAHs were derived from the primary diagnosis leading to the hospital admission. The PAH calculus is described in detail elsewhere^15^. The prescription of me-too drugs was determined by the “Pharmazentralnummer” (PZN) of prescribed drugs. The PZN serves as a unique identifier for a specific pharmaceutical preparation. Me-too drugs can be by identified unambiguously the PZN.

These five key indicators have been examined since the inception of the GPCHC evaluation.

### Data analysis

Descriptive analyses were conducted for patient characteristics in the GPCHC and control cohort (regular care). In order to assess the association between GPCHC and the key indicators, we used multivariable Poisson regression models for our count data. Potential clustering effects of patients within GP practices were addressed using Generalized Estimating Equations (GEEs). GEEs models were also used for our longitudinal 12-years-models in order to regard autocorrelation of individuals over time. Patient age, gender, nationality, morbidity, nursing care level, and nursing home residency were included into our multivariable models in order to adjust for those variables. The morbidity was measured by the so called “Carlson-Index” (CI). Based on the ICD-10 diagnoses codes, it was possible to determine the CI-Score in order to approximate patients’ overall morbidity. There are particular diagnoses corresponding to more severe conditions. Values between 1 and 6 are assigned for those diagnoses. Finally, a sum score is determined for each individual. The underlying calculus is described in detail elsewhere^18^.

The models’ goodness of fit was evaluated using the quasi-likelihood under the independence model criterion (QIC), with the most fitting models preferred. Results with P-values below 0.01 were considered statistically significant. In order to adjust for multiple testing, we applied the conservative Bonferroni Method.

Data storage and extraction were performed using MySQL Community Server x64 (Oracle Corporation, Redwood Shores, CA, USA). Statistical analyses were conducted using SAS 9.4 (SAS Institute Inc., Cary, North Carolina, USA).

### Ethics approval and consent to participate

Ethics approval was provided by the Heidelberg University Ethics Committee (No. S-359/2013). All patients provided written informed consent for participation in the GPCHC programme. Patients in the regular care cohort were extracted from an anonymized database of AOK claims data

We confirm that all methods were carried out in accordance with relevant guidelines and regulations.

## Results

Overall 1,929,957 patients were included in our cross sectional analyses for 2022. For the longitudinal analyses 3,834,372 patient years were drawn upon from 2011 to 2022 (12 years). A patient-year is defined as the observation of one patient for a period of one calendar year.

### Demographics

Table 1 presents the sociodemographic characteristics of the GPCHC and the regular care group for the observation year 2022. The mean age of GPCHC individuals in 2022 was approximately 55 years, compared to about 52 years in the regular care group. The gender distribution was nearly identical in both groups (approximately 54% female and 46% male). The burden of disease (morbidity), measured by the Charlson Comorbidity Index, was higher in the GPCHC group (1.37) than in the regular care group (1.01). This higher disease burden among GPCHC individuals must be regarded as clinically relevant. The mean duration of GPCHC participation among the study population was 8.7 years.

**Table 1:** Patient demographics.

	Observation Year 2022 Patients included (*n* = 1,929,957)	
	GPCHC Patients (*n* = 1,255,445)	Regular Care Patients (*n* = 674,512)
Age ± SD (in 2022)	55.20 ± 18.97	52.33 ± 19.48
Gender male female	45.92% 54.08%	46.32% 53.68%
Nationality German other	78.97% 21.03%	76.88% 23.12%
Insurance Status Policyholder Dependent Retiree	58.84% 6.59% 34.56%	62.87% 7.12% 29.02%
Morbidity ± SD based on Charlson-Index	1.37 ± 2.05	1.01 ± 1.76
Duration of GPCHC enrollment Quarters ± SD	34.60 ± 16.43	-

**Table 2:** Cross sectional results (2022).

	Observation Year 2022 Patients included (*n* = 1,929,957)			
	CPCHC Patients Ø ± SD *n* = 1.255.445	Regular Care Patients Ø ± SD *n* = 674.512	Adjusted Difference (SE) [95%-CI] CPCHC vs. Regular Care	Difference in % Significance – adjusted –^**^ CPCHC vs. Regular Care
GP-contacts Average number per patient	13.27 ± 10.86	8.43 ± 12.22	+ 5.08 (0.047) [4.985; 5.169]	+ 25.47% *p* < 0.0001 [24.99%; 25.92%]
Uncoordinated consultations with a non-GP-specialist Average number per patient	1.95 ± 6.06	3.29 ± 9.04	−1.48 (0.114) [−1.708; −1.263]	−38.85% *p* < 0.0001 [−44.84%; −33.15%]
All-cause hospitalizations Average number per patient	0.224 ± 0.671	0.216 ± 0.661	−0.021 (0.004) [−0.023;−0.019]	−8.61% *p* < 0.0001 [−9.43%; −7.79%]
Potentially avoidable hospitalizations Average number per 100 patients	14.34 ± 32.46	14.01 ± 32.16	−0.506 (0.001) [−0.771; −0.240]	−2.46% *p* < 0.0001 [−3.75%; −1.17%]
Prescription of me-too drugs Prescriptions at the GP, share in %	1.56 ± 6.80	2.11 ± 8.65	−0.666 (0.014) [−0.693; −0.639]	−24.82% *p* < 0.0001 [−25.83%; −23.81%]

### Cross Sectional Results

Table 2 presents the results of the cross-sectional analyses for the year 2022. Columns 2 and 3 report unadjusted mean values for GPCHC and regular care individuals. Columns 4 and 5 display the adjusted absolute and relative differences of GPCHC individuals compared to regular care individuals, as well as the type I statistical error (p-value).

For all five key indicators, the results are statistically significant (*p* < 0.0001) in favor of GPCHC individuals. The percentage differences are particularly pronounced for GP contacts (+ 47.46%), uncoordinated consultations with a non-GP specialist (− 38.85%), and prescription of me-too drugs (− 24.82%). However, the observed percentage differences for all-cause hospitalizations (− 8.61%) and potentially avoidable hospitalizations (− 2.46%) must also be considered systemically relevant in light of the high costs associated with inpatient care.

### Longitudinal Results

**Fig. 1:**
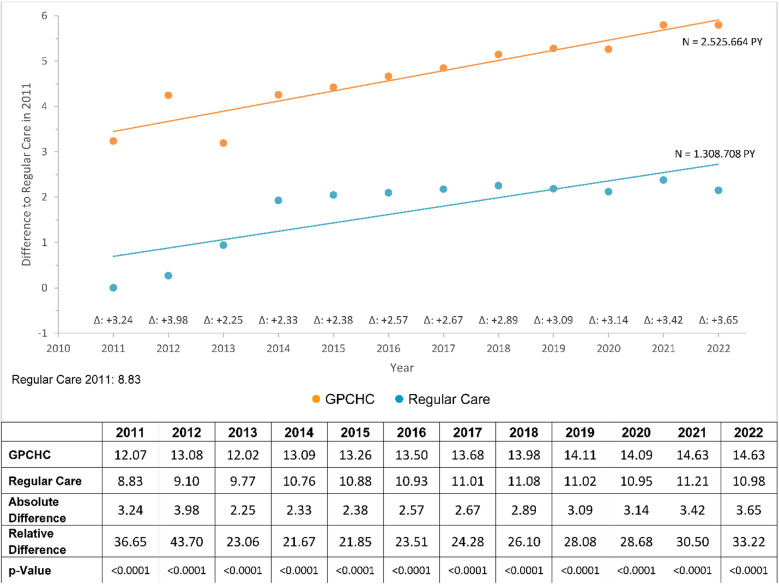
GP-contacts, observation period from 2011 to 2022. Values adjusted for covariables ( GPCHC,  Regular Care).

Our cross sectional observations were confirmed by longitudinal data for the period 2011 to 2022. In the longitudinal analysis of “GP contacts,” the relative difference persisted between 21% and 37% across all study years (Fig. 1).

**Fig. 2:**
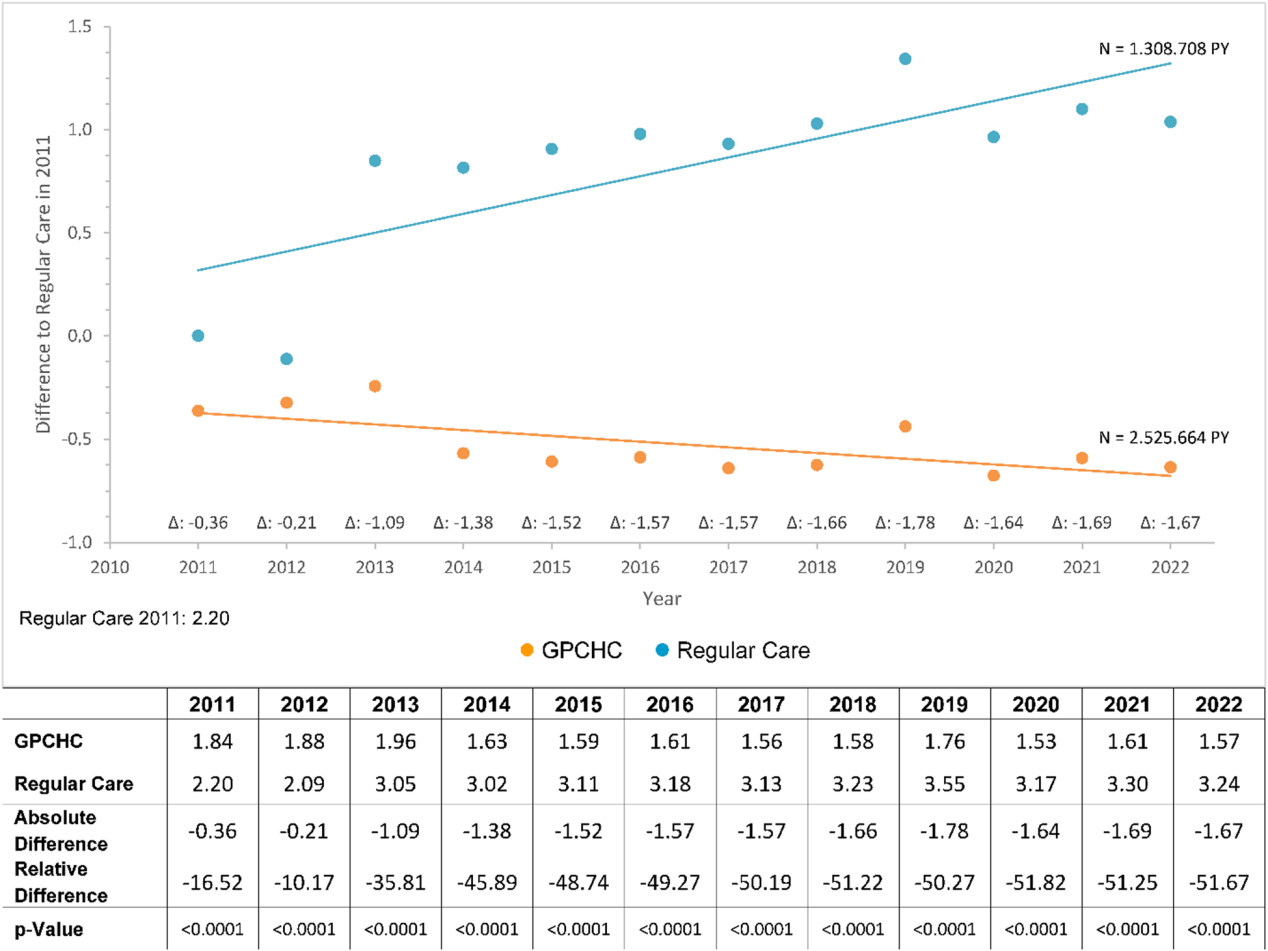
Uncoordinated consultations with a non-GP-specialist. Uncoordinated consultations with a non-GP-specialist, observation period from 2011 to 2022. Values adjusted for covariables ( GPCHC,  Regular Care).

Across the entire observation period, CPCHC individuals consistently exhibited fewer “uncoordinated specialist consultations.” The difference of 35.8% in favor of the CPCHC group in 2013 increased to more than 45% in the following year and reached 51.7% in 2022 (Fig. 2). Considering the longitudinal data on GP contacts and uncoordinated specialist consultations for 2022, CPCHC individuals had, on average, more than three additional GP contacts compared with regular care individuals, while at the same time exhibiting markedly more than one fewer uncoordinated specialist consultation. Given the large number of insured individuals included over the observation period, these differences can be regarded as highly relevant for overall healthcare provision.

**Fig. 3:**
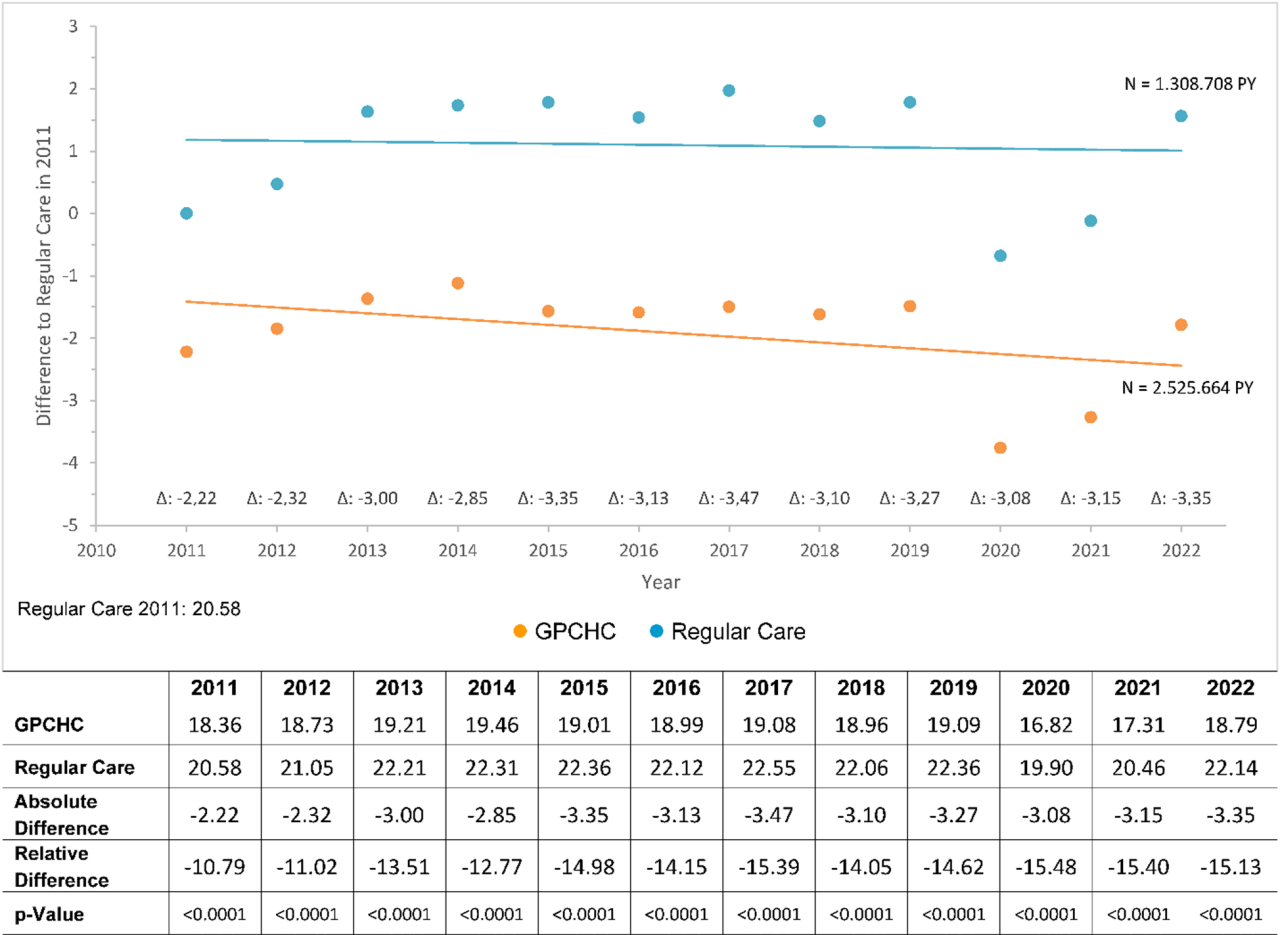
All-cause hospitalizations. All-cause hospitalizations, observation period from 2011 to 2022. Values adjusted for covariables ( GPCHC,  Regular Care).

With respect to “all-cause hospitalizations” (per 100 individuals) the longitudinal analysis revealed percentage differences ranging from − 15.5% to − 10.8% in favor of GPCHC individuals (Fig. 3). Compared with the cross-sectional analysis, larger differences were observed.

**Fig. 4:**
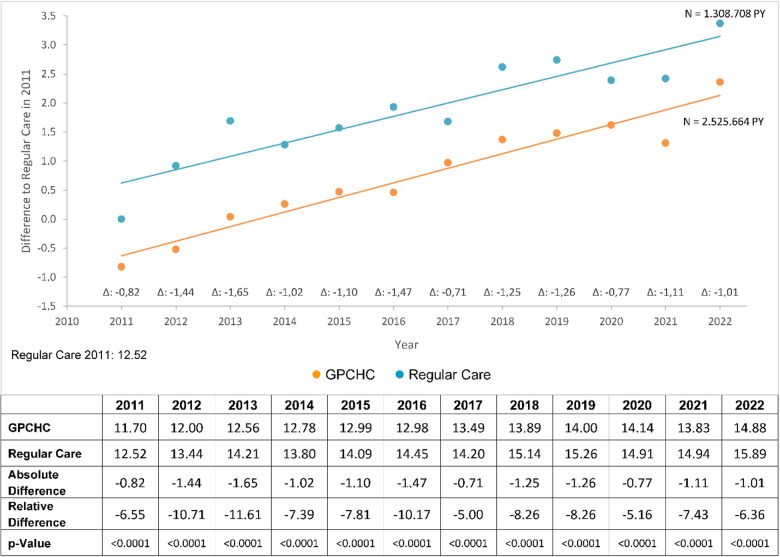
Potentially avoidable hospitalizations. Potentially avoidable hospitalizations, observation period from 2011 to 2022. Values adjusted for covariables ( GPCHC,  Regular Care).

With respect to potentially avoidable hospitalizations (per 100 hospitalized individuals), the longitudinal analysis showed statistically significant lower admission rates for GPCHC individuals (Fig. 4). On average, the absolute difference was close to 1, corresponding to roughly one additional avoided hospitalization per 100 individuals compared with the regular care group.

**Fig. 5:**
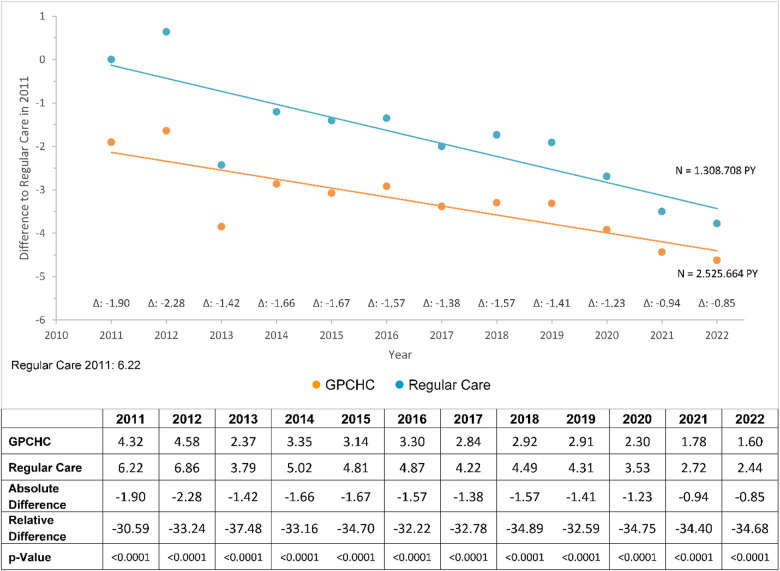
Prescription of me-too drugs. Prescription of me-too drugs, observation period from 2011 to 2022. Values adjusted for covariables ( GPCHC,  Regular Care).

With respect to the share of “prescriptions of me-too drugs” among all prescriptions issued by general practitioners, the longitudinal analysis revealed substantially fewer “me-too drug” prescriptions for HZV individuals. The relative difference ranged between 30.5% and 37.5% (Fig. 5).

Although the absolute differences are not large, when considered in the context of a very large insured population, these differences are of clear health-economic relevance.

## Discussion

Our 2025 evaluation of the GPCHC program in Baden-Württemberg reaffirms the model’s effectiveness in improving key health outcomes. Compared to non-enrolled individuals, GPCHC participants benefit from enhanced care coordination, reduced hospital utilization, and more appropriate medication use. Cross-sectional analyses showed significant benefits for several indicators, which were confirmed by longitudinal data for the period 2011 to 2022. A consistent pattern of favorable associations emerged over time, with a sustained or even increasing magnitude of positive differences in clinical and utilization outcomes.

Particularly cost-relevant outcomes -such as those associated with pharmacotherapy and inpatient care- demonstrated pronounced longitudinal effects. The reduction in PAHs and medication-related risks^17^ underscores the model’s contribution to system efficiency and patient safety.

These findings are consistent with international evidence indicating that longitudinal, coordinated primary care -such as in GPCHC- can reduce the burden of inappropriate polypharmacy, particularly among patients with chronic conditions^19–21^. Integrated primary care models have also demonstrated improvements in clinical indicators, including better control of hypertension, diabetes, and reduced stroke incidence^20,22^. Furthermore, structured medication reviews implemented within primary care settings have proven effective in enhancing medication safety in polypharmacy contexts^23^. Such international comparisons further validate the German GPCHC model: countries with well-established primary care systems -such as the Netherlands, the United Kingdom, and the Nordic states- consistently report reduced avoidable hospitalizations, higher continuity of care, and improved patient satisfaction within gatekeeping and team-based frameworks^4,5,8,24^. The five-year survival advantage observed in GPCHC cohorts’ mirrors findings from UK-based models of patient-centered medical homes^11,25^. Additionally, improved medication safety and lower prescription rates of fall-risk-increasing drugs (FRIDs) among GPCHC participants align with global evidence highlighting the importance of continuity and interdisciplinary care in the management of elderly and multimorbid populations^17,26^.

Collectively, these results highlight the potential of structured primary care programs not only to maintain but also to expand their impact in settings marked by rising chronic disease burden and healthcare complexity.

Spending on health and, more specifically, on primary care varies significantly across countries with GP-centered systems. For example, the Netherlands allocates 14–16% of its total health budget to primary care, with overall health expenditure amounting to 10.7% of GDP. Denmark and Sweden maintain similar ratios, with Denmark investing 15% in primary care and 10.1% of GDP in total health spending. These levels of investment are associated with improved continuity, effective chronic care coordination, and high patient satisfaction^25,27^.

The United Kingdom, despite pressures on the NHS, maintains primary care spending at about 12–14% and total health expenditure at 12% of GDP, supporting robust performance in chronic disease management and integrated care^28^.

Germany also spends around 12.8% of its GDP on healthcare but has traditionally allocated only 6–8% to primary care^29^. However, this situation is evolving. The GPCHC model now covers over three million people in Baden-Württemberg and more than ten million nationwide^30^.

Although Germany still lags some comparator countries in terms of investment in primary care, the progress observed in the context of GPCHC emphasizes its potential. Expanding the scope of care, harmonizing funding priorities and strengthening structural reforms could help to move Germany towards a more integrated and efficient primary care system.

In the United States, patient-centered medical home (PCMH) models mirror structural elements of the German GPCHC program, emphasizing team-based care, data-driven chronic disease management, and care continuity. A five-year evaluation by Blue Cross & Blue Shield of Rhode Island reported a 5% reduction in total healthcare costs, a 16% lower hospitalization and ED visit rate among chronically ill patients, and 30% fewer readmissions by year five in PCMH settings compared to traditional care models^31^. Similarly, a Maryland study found that PCMH enrollment among high-cost privately insured adults led to a 34% reduction in the likelihood of remaining in the highest cost category, with improved continuity of care and reduced hospitalizations^32,33^. These findings aligned with earlier evaluations show that PCMH implementation can achieve measurable reductions in hospital utilization, improved outcomes in chronic conditions like diabetes and hypertension, and overall cost containment^34–37^. While early results showed some variability, more recent longitudinal evidence highlights that mature PCMH implementations deliver sustained system-level improvements. Taken together, this growing U.S. literature confirms that structured, GP-equivalent primary care models -when properly implemented- can reduce emergency and inpatient care, improve chronic disease management, and support cost-effective care delivery. The international convergence of evidence underscores the value of continued investment in robust primary care infrastructure.

Despite the use of comprehensive administrative data and rigorous risk adjustment, the observational design of this study precludes definitive causal inference. Residual confounding cannot be fully excluded, particularly due to potential selection effects, such as patient self-enrollment based on care-seeking preferences or differences in physician engagement and motivation. Voluntarily participating patients may exhibit greater health awareness and/or higher levels of compliance compared to those who do not take part. This may introduce a potential selection bias, but our data set does not allow to adjust for these – potentially different – behavioral patterns. However, the robustness of findings across multiple outcomes, time periods, and analytic methods strengthens the credibility of the results.

When situated within the international context, GPCHC outcomes mirror those reported in countries with strong primary care systems—such as the Netherlands, Denmark, and the United Kingdom—where gatekeeping, team-based care, and higher primary care investment contribute to reduced inequities and improved population health. Similar trends are also evident in the United States, where patient-centered medical home models have demonstrated reductions in hospitalizations and emergency visits, better chronic disease management, and cost savings when consistently implemented.

While Germany continues to allocate a comparatively lower share of its healthcare spending to primary care, the positive outcomes achieved within the GPCHC framework demonstrate that targeted structural reforms can yield substantial benefits even under constrained investment conditions.

The findings reaffirm global evidence linking greater investment in primary care with better clinical outcomes, fewer avoidable hospital admissions, and more equitable service access. In this regard, the GPCHC model offers not only successful regional implementation but also a scalable and transferable framework for primary care reform in high-income healthcare systems facing demographic change, chronic disease burdens, and financial pressures.

## Data Availability

The AOK Baden-Wuerttemberg can be contacted for access to the claims data. Data are available on reasonable request.
